# Hemispheric Asymmetry and Task Accuracy

**DOI:** 10.21203/rs.3.rs-6495742/v1

**Published:** 2025-05-13

**Authors:** Dardo Tomasi, Nora Volkow

**Affiliations:** National Institutes of Health; National Institute on Drug Abuse National Institutes of Health

## Abstract

Interhemispheric asymmetry is a core feature of human brain organization, yet its functional relevance across cognitive domains remains incompletely understood. Using data from 989 participants in the Human Connectome Project, we examined patterns of functional asymmetry and their relationship to task performance across seven domains—motor, language, social cognition, relational processing, working memory, gambling, and emotion. An fMRI-derived asymmetry index was computed across 17 task contrasts and mapped onto the cortical surface. Both fMRI signal amplitude and asymmetry were positively associated with task accuracy across multiple networks and cognitive domains. These associations were strongest in language, frontoparietal, and dorsal attention networks during high-demand tasks, such as story comprehension, relational processing, and working memory. Partial least squares regression revealed that while amplitude was the more robust predictor of task accuracy, asymmetry contributed unique, complementary variance. These findings suggest that greater neural activation and stronger hemispheric differentiation jointly support better cognitive performance. Together, our results underscore the behavioral relevance of both fMRI signal amplitude and lateralization, offering new insights into the functional architecture and efficiency of the human brain.

## INTRODUCTION

Interhemispheric differences in brain function, also known as brain asymmetry, are of long-standing interest in neuroscience due to their critical role in cognitive processes like language, attention, and motor control^1–6^. Functional asymmetry, the unequal activation of brain regions between the hemispheres during cognitive tasks, is believed to enable different brain areas to specialize in distinct functions, promoting efficient information processing^7,8^. For example, the left hemisphere is involved in language tasks^9^, and relational processing, which involve integration and manipulation of complex information that primarily engages the left anterior prefrontal cortex^10,11^. In contrast, the right hemisphere often dominates in spatial attention, facial recognition, and emotional processing tasks^12,13^. A recent study have shown that healthy adolescents/young adults with left-hemisphere language dominance (n = 39) often display this pattern of asymmetry, and those with atypical right-hemisphere language dominance (n = 24) tend to show a fully mirrored organization^14^. Importantly, in that study, individuals whose asymmetry patterns deviated from the typical segregation in more than one domain exhibited poorer cognitive performance. Yet, the generalizability and reproducibility of these findings remain unclear.

Functional asymmetry reflects the brain’s tendency to lateralize cognitive processes, a feature thought to support neural specialization and processing efficiency^3,15^. Evidence suggests that individuals with greater task-related asymmetry may have higher accuracy, consistent with the notion that lateralized activation patterns reflect the optimized recruitment of specialized cortical systems^16^. Consistent with this notion, a recent large-scale meta-analysis found that regions exhibiting greater lateralization tend to have weaker interhemispheric structural connectivity^17^. This suggest that regions that are more lateralized may rely less on costly interhemispheric integration and instead engage more efficient unilateral processing during cognitively demanding tasks.

The lateralized role of the hemispheres also manifests itself clinically such that patients suffering from left hemisphere lesions are more likely to exhibit language impairments (aphasia) whereas patients suffering from right hemisphere lesions are more likely to exhibit abnormal emotional and social behaviors^18,19^. This has led to the development of models of emotional lateralization that identify right hemisphere’s structures involved with the computation, communication, modulation, and experience of emotions^20–22^. Functional brain asymmetry is highly relevant to psychiatry as many disorders involve disruptions in the typical balance of hemispheric activity^23^. Individuals with depression, for example, often show reduced left-hemisphere activity associated with positive affect and reward processing, alongside increased right-hemisphere activity related to negative emotions^24^. Similar disruptions have been observed in schizophrenia^25–27^, where atypical asymmetry patterns may contribute to deficits in language^28^, executive function^29^, and social functioning^30^. Additionally, reductions in asymmetry occur naturally with aging, which may impact cognitive flexibility and adaptability^31,32^. Understanding these patterns of asymmetry could therefore provide a foundation for targeted interventions, such as neuromodulation, to optimize hemispheric balance.

The functional significance of hemispheric specialization and its variability across individuals are unclear^33–35^. While greater lateralization has traditionally been associated with more efficient neural processing in healthy individuals^36^, reduced hemispheric asymmetry in patients has been interpreted in divergent ways. One perspective proposes that lower specialization may reflect compensatory recruitment^37^, wherein bilateral activation serves as an adaptive mechanism to support performance^38^, particularly under increased cognitive demand or in aging populations^39^. According to this view, reduced asymmetry is beneficial and predicts better behavioral outcomes^38^. In contrast, the dedifferentiation hypothesis^40^ suggests that decreased hemispheric specialization results from a failure to suppress irrelevant or task-inappropriate activation in the contralateral hemisphere, leading to less efficient processing and poorer performance. These opposing models highlight the importance of examining not only the presence of asymmetry, but also its relationship to task-evoked brain activity and behavioral performance. Furthermore, although asymmetry appears to optimize brain efficiency, it is still unclear how deviations from typical patterns impact task performance^41^. Moreover, the literature on functional asymmetry yields highly variable findings, complicating systematic comparisons of asymmetry indices across studies^42^.

Surface-based analyses offer a precise approach for studying brain asymmetry, particularly because they focus on the cortical sheet where most higher-order cognitive functions occur^43^. Unlike traditional volumetric methods, surface-based approaches rely on symmetrical templates for the left and right cortical sheets, allowing for more accurate alignment of cortical features across individuals, minimizing anatomical variability and enhancing the detection of subtle asymmetry patterns^44^. This precision is particularly beneficial for studies examining lateralized activation across cortical regions.

The Human Connectome Project (HCP; https://www.humanconnectome.org/), a large-scale publicly available imaging dataset, provides high-quality, standardized fMRI data in surface space. This dataset’s diversity and quality make it ideal for examining asymmetry across various cognitive tasks and demographic groups. Leveraging the HCP task-fMRI data, our study aims to explore how lateralized brain activity varies across cognitive domains, using a threshold-independent method for calculating the asymmetry index. We also aim to examine the role of functional asymmetry in task performance. We hypothesized that task accuracy would be positively associated with both greater fMRI signal amplitude and greater hemispheric asymmetry, particularly in cognitively demanding tasks, consistent with more efficient neural engagement^45^. In parallel, we hypothesized that distinct cognitive domains would exhibit reproducible patterns of functional asymmetry, with left-hemisphere dominance for language and relational processing, contralateral patterns for motor tasks, right-hemisphere dominance for emotion and social cognition, and more balanced patterns for working memory and gambling tasks. Asymmetry was expected to be most pronounced in the somatomotor (SMM) and language (LAN) networks.

## RESULTS

**Figure 1:**
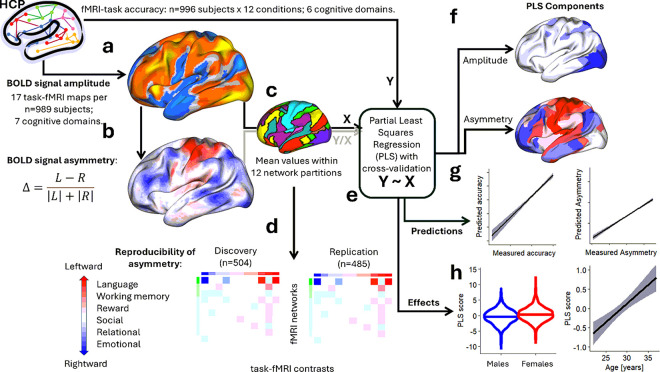
Study Flow Chart. fMRI contrasts across 17 task epochs were analyzed for 989 healthy young adults from the Human Connectome Project (HCP; **a**). Left (L) and right (R) hemisphere fMRI signals were assessed independently at 32,492 cortical vertices per participant and contrast to quantify functional interhemispheric asymmetry, D using Eq [1] (**b**). Individual maps of fMRI signal amplitude and asymmetry were averaged across 12 predefined network partitions^46^ for each participant and contrast (**c**). The sample was divided into Discovery (n=504) and Replication (n=485) subsamples to examine the reproducibility of the functional asymmetry index. Functional networks were ranked by root-mean-square values of asymmetry, and task contrasts by mean asymmetry values across participants within each subsample (**d**). Partial least squares (PLS) regression was applied to extract principal components of fMRI signal amplitude and asymmetry (**e**). These components were used to predict asymmetry from fMRI signal amplitude, and task accuracy independently for amplitude and asymmetry (**f**) and assessed the effects of age and sex on PLS regression scores from amplitude and asymmetry (**g**).

We utilized fMRI datasets from the publicly available WU-Minn HCP 1200 Subjects data release (http://www.humanconnectome.org/). A total of 989 participants, each of whom completed seven fMRI tasks (motor, language, social cognition, relational processing, working memory, gambling, and emotion) with root-mean-square (RMS) head displacement < 2mm, were included (Fig. 1a).

We analyzed 16,853 individual fMRI signal contrasts corresponding to 17 conditions per participant, involving 91,281 grayordinates within the brain^47^. A functional asymmetry index, D, the normalized difference between left (L) and right (R) hemisphere fMRI signal amplitudes^43^,

$$\Delta =\frac{L\;-\;R}{\left|L|\;+\;|R\right|}\;,$$

and the mean bilateral amplitude of the fMRI signal,

$$A=\frac{\left|L|\;+\;|R\right|}{2}\;,$$

were calculated and mapped to the 32,492 vertices of the left cortical hemisphere for each contrast independently (Fig. 1b). To evaluate the reproducibility of the asymmetry index, we divided the participants into Discovery (n = 504) and Replication (n = 485) subsamples, while matching these cohorts by age, sex, and body mass index (BMI) using the caTools R-library (https://cran.r-project.org/web/packages/caTools/). There were no significant differences in sex, age, race, BMI, handedness, and task accuracy between Discovery and Replication subsamples (Supplementary Table S1).

### Vertex-wise interhemispheric asymmetry

**Figure 2:**
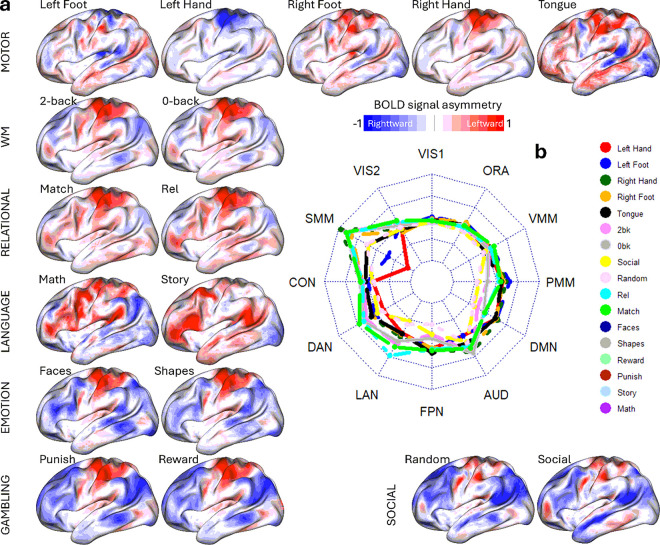
Functional Asymmetry Maps. Vertex-wise asymmetry maps for 17 epochs of 7 fMRI tasks rendered on the left cortical hemisphere (a). The radar chart (b) summarizes average asymmetry for each task epoch across 12 network partitions: Visual (VIS1 and VIS2), Somatomotor (SMM), Cingulo-Opercular (CON), Dorsal Attention (DAN), Language (LAN), Frontoparietal (FPN), Auditory (AUD), Default-Mode (DMN), Posterior (PMM) and Ventral Multimodal (VMM), and Orbito-Affective (ORA) networks. Sample includes 989 healthy young adults. One-sample t-test. P<0.05, FDR-corrected for multiple comparisons. Radar chart inner and outer limits are set at −0.3 and 0.3, respectively.

fMRI signal asymmetry was task-specific (Fig. 2 and Supplementary Figure S1; P < 0.05, FDR-corrected). In the motor task, a one-sample t-test revealed opposing interhemispheric asymmetry, primarily in the somatomotor (SMM) cortex: left hand/foot movements showed greater activation in the right hemisphere, while right hand/foot movements activated the left hemisphere more. For the language task, story listening—compared to solving auditory arithmetic problems—showed greater leftward asymmetry in the language (LAN), frontoparietal (FPN), and default mode networks (DMN), with rightward asymmetry in the visual and SMM cortex. A similar asymmetry pattern appeared when contrasting relational (relation vs. match epochs) or social (social vs. random) task epochs. Higher working memory load (2-back > 0-back) was associated with leftward asymmetry in visual and SMM cortex and rightward asymmetry in DMN regions.

In the emotion task, matching faces versus shapes was linked to predominant rightward asymmetry in the dorsal attention (DAN), FPN, and posterior multimodal (PMM) networks. For the gambling task, asymmetry differences between reward and punish epochs were minimal. The radar plot in Fig. 2 visually summarizes these asymmetry trends in the functional networks for the various tasks. The asymmetry patterns reproduced in Discovery and Replication subsamples (Figure S2 and Supplementary Statistical results in CIFTI format).

### Networks and tasks ranked by asymmetry

Asymmetry patterns were averaged within 12 network partitions^46^, for each participant and fMRI contrast (Fig. 1c). To determine the degree of asymmetry by network, networks were ranked based on the RMS of asymmetry across contrasts. To evaluate asymmetry by contrast, contrasts were ranked by their mean asymmetry across networks (Fig. 1d).

**Figure 3:**
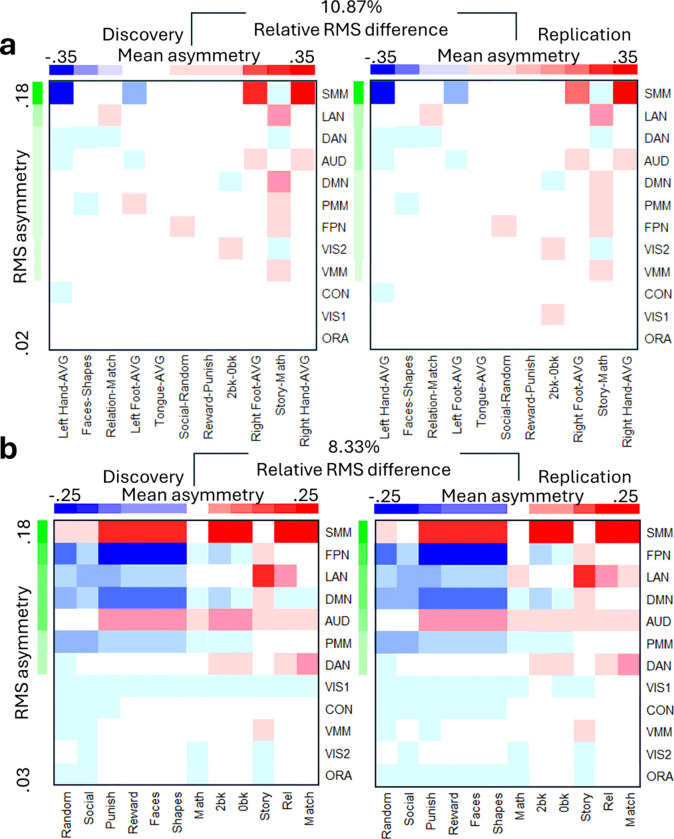
Network asymmetry. Average asymmetry within 9 functional networks is ranked by root-mean square (RMS) values and by mean asymmetry across 11 task contrasts (a) or 12 task epochs (b), independently for the Discovery (n=504) and Replication (n=485) subsamples. Network abbreviations: VIS1 and VIS2, visual; CON, cingulo-opercular, DAN, dorsal-attention; SMM, somatomotor; FPN, frontoparietal; AUD, auditory; DMN, default-mode; PMM and VMM, Posterior and Ventral Multimodal; ORA, orbito-affective.

Across task contrasts, interhemispheric asymmetry was most pronounced in the SMM network (Figs. 3a and 3b; P < 0.05, FDR-corrected), revealing a reproducible yet complex pattern. For example, relative to the overall asymmetry induced by the motor task, movements of the left hand/foot were linked with rightward asymmetry in the SMM, DAN, and auditory (AUD) networks, while movements of the right hand/foot showed the opposite pattern (Fig. 3a). In the language task, story epochs—compared to math epochs—showed rightward asymmetry in the SMM, DAN, and visual (VIS1 and VIS2) networks, and leftward asymmetry in LAN, AUD, DMN, PMM, FPN, and ventral multimodal (VMM) networks (Fig. 3a). Across all task epochs, the SMM and AUD networks exhibited consistent leftward asymmetry except for rightward asymmetry with movements of left hand/foot and the DMN exhibited consistent rightward asymmetry, except for the story epoch of the language task (Fig. 3b). Differently, FPN and LAN networks displayed notable rightward asymmetry during social (random and social), gambling (punish and reward), emotion (faces and shapes), and working memory (0-back and 2-back) task epochs, with a marked leftward asymmetry during the story epochs of the language task (Fig. 3b).

### Association between fMRI signal amplitude and asymmetry

**Figure 4:**
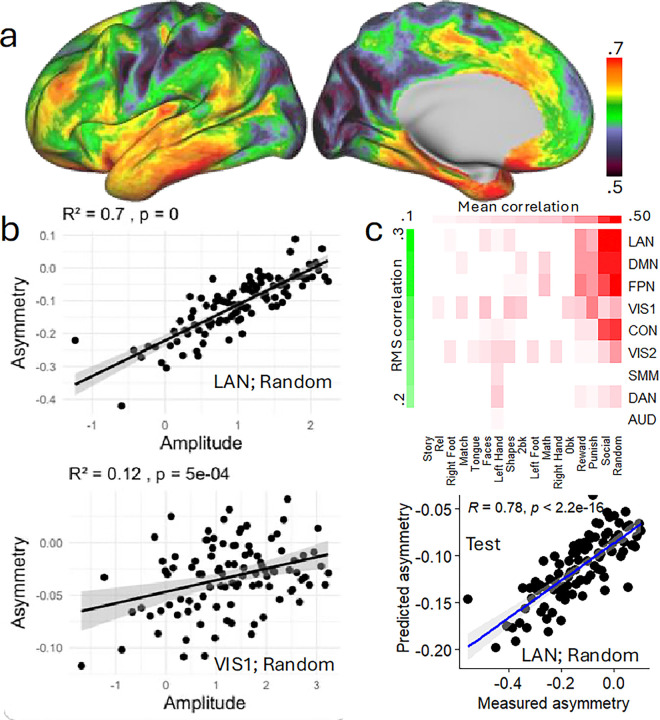
Linear association between fMRI signal asymmetry and amplitude. (**a**) Average correlation between hemispheric asymmetry and BOLD signal amplitude across 17 task epochs, rendered on the lateral (left) and medial (right) surfaces of the left cortical hemisphere. (**b**) Representative scatter plots illustrating the linear relationship between asymmetry and amplitude in the language (top) and primary visual (bottom) networks during the random epoch of the social cognition task, which was the task epoch that showed the strongest association. Each point reflects the average of 10 individuals grouped by similar fMRI amplitude. (**c**) Partial least squares (PLS) prediction of asymmetry based on mean bilateral BOLD amplitude across 17 task contrasts and 9 major networks. The top matrix shows correlations between predicted and observed asymmetry, ranked by mean values across epochs (columns) and by root-mean-square values across networks (rows), thresholded at P < 0.05 (uncorrected). The bottom scatter plot highlights prediction performance for the language network during the random epoch. Network abbreviations: VIS1 and VIS2, visual; CON, cingulo-opercular; DAN, dorsal attention; SMM, somatomotor; FPN, frontoparietal; AUD, auditory; DMN, default mode. Sample: 989 healthy adults.

To identify spatial patterns linking signal amplitude and functional asymmetry across the cortex, we performed vertex-wise correlations between these two measures and averaged the resulting maps across all 17 task epochs. This approach was designed to reduce task-specific noise and highlight consistent, task-general associations between amplitude and asymmetry. The strongest positive associations (R > 0.65; corrected Cohen’s *d* > 0.54) were observed in lateral occipital, ventral temporal, inferior frontal, and inferior parietal regions (Fig. 4a), suggesting that greater neural engagement in these areas is consistently linked to increased asymmetry. In contrast, weaker correlations (R < 0.55; *d* < 0.41) were found in medial prefrontal, superior frontal, and medial parietal cortices, including regions of the DMN, where amplitude and asymmetry appeared more modestly coupled. These findings indicate that the amplitude-asymmetry relationship varies systematically across cortical territories, with stronger associations in regions typically implicated in lateralized cognitive functions such as language and executive control.

To also examine the relationship between asymmetry and the amplitude at the network level, we first calculated for each individual, the mean fMRI signal amplitude within nine major networks, separately for the left and right hemispheres and each of the 17 task epochs (Figure S1 and Supplementary Statistical Results in CIFTI format). Individuals were then ranked based on their average bilateral amplitude and this ranking was used to form 100 groups of 10 individuals with similar amplitude levels, independently for each task epoch and network. Then for each group, task epoch, and network, we computed the average amplitude and average asymmetry to assess the relationship between these metrics at a group level.

The correlation analysis revealed that the amplitude-asymmetry association across participants was strongest for the LAN (R = 0.896 for the match epoch, left hemisphere; and 0.884 for the random epoch, right hemisphere; *d* > 1.2; Figure S 3). The association was weakest for the story epoch, bilaterally in the VIS2 (R = 0.21; *d* = .14).

### fMRI signal amplitude predicts asymmetry

We applied partial least square (PLS) regression to investigate whether the mean interhemispheric fMRI signal amplitude predicts asymmetry (Figs. 1e and 1g). PLS is designed to handle scenarios where predictors (e.g., mean amplitudes) are highly correlated across networks and contrasts (multicollinear)^48^ as in this study (Figure S4). Different to multiple linear regression, which can lead to unstable or overfitted models, PLS reduces the data’s dimensionality by constructing orthogonal components that maximize the covariance between predictors and the response variable (e.g., asymmetry)^49^. A total of 153 predictors (mean fMRI signal amplitude values within 9 networks for 17 task epochs) were used to estimate 153 response variables (mean asymmetry values within 9 networks for 17 task epochs) using 12 PLS components with within-sample 10 -fold cross-validation. These components captured 63% of the variance, providing robust predictions without overfitting.

To evaluate the robustness of PLS predictions in new samples, we trained the PLS model using within-sample 10-fold cross-validation in the Discovery sample, then applied this optimal model to predict asymmetry from amplitudes in the Replication sample (Figures S5a and S5b). To complete the 2-fold cross-validation, we reversed the process: training the PLS model in the Replication sample and using it to predict asymmetry from amplitude in the Discovery sample (Figures S5c and S5d). The 2-fold cross-validation across test subsamples was successful, reproducing the same prediction patterns observed in the training.

PLS regression revealed a complex predictive pattern. For epochs within the social and gambling tasks, the amplitude regressors successfully predicted asymmetry primarily in the LAN, DMN, FPN, and cingulum-opercular (CON) networks, with lesser contributions from VIS1, VIS2, and DAN (Fig. 4c; P < 0.05, Bonferroni-corrected). Amplitude regressors also predicted asymmetry in VIS1 and VIS2 for working memory, relational, and emotion task epochs, as well as in VIS2 for motor task epochs (P < 0.05, Bonferroni-corrected). Further predictions were seen in DMN and FPN for the math epoch (P < 0.05, Bonferroni-corrected).

### Task accuracy

**Figure 5:**
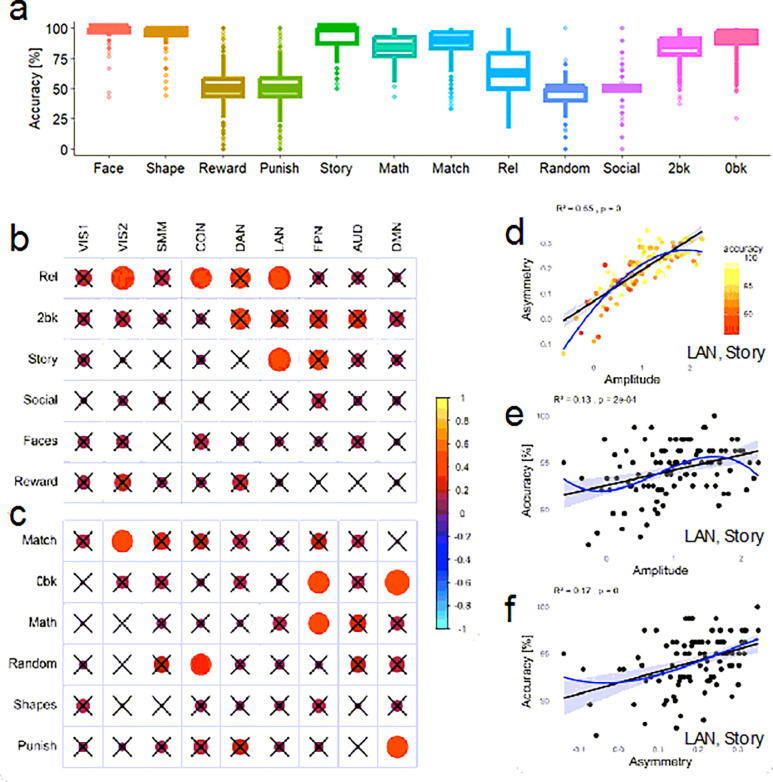
Task accuracy and asymmetry. (**a**) Boxplot representing the distribution of performance accuracy across 12 main task epochs. The box spans the interquartile range (IQR), containing the middle 50% of data points and the line within each box denotes the median. Whiskers extend from each box to the smallest and largest values within 1.5 times the IQR from the first and third quartiles. Data points beyond the whiskers are displayed as outliers, representing values outside this range. Corrplot showing Pearson correlations between accuracy and fMRI signal asymmetry for 9 major networks and for task (b) and control (c) epochs. Non-significant correlations (p≥0.001) are marked with a black cross. The color of each circle represents the direction and strength of the correlation, while the area of each circle is proportional to the value of the correlation coefficient, reflecting its magnitude. (**d-f**) Representative scatter plots illustrating linear relationships between accuracy (color coded in d) and both activation amplitude (e) and hemispheric asymmetry (f) within the language network during the story epoch of the language task. Each point reflects the average of 10 individuals grouped by similar amplitude. Black and blue lines are 1^st^ and 3^rd^ order polynomial fits to the data. The shaded area represents the 95% confidence interval around the fitted line. Network abbreviations: VIS1 and VIS2, visual; CON, cingulo-opercular; DAN, dorsal attention; SMM, somatomotor; FPN, frontoparietal; AUD, auditory; DMN, default mode. Sample: 989 healthy adults.

Participants demonstrated high overall accuracy across the task-fMRI paradigms (Fig. 5a and Table 1). Accuracy was calculated for each individual and task based on correct responses during scanner-based performance. For the emotion, language, relational, and working memory tasks, the mean accuracy was above 60%, indicating good task engagement and compliance. In contrast, average accuracy fell chance level (~ 50%) for both the gambling and social cognition tasks, reflecting either the probabilistic nature of task outcomes or limitations in performance measurement in those paradigms. Note that the motor task was designed primarily to map robust sensorimotor activation rather than assess performance variability, and therefore does not include accuracy metrics, as the simple, cued movements are expected to elicit high compliance with minimal behavioral variance.

We next assessed whether task accuracy was associated with the amplitude and asymmetry of fMRI responses across cognitive domains. As shown in Fig. 5b–c, greater signal amplitude and asymmetry were significantly correlated with better accuracy in multiple networks and task epochs (P < 0.05, Bonferroni-corrected). Notably, these effects were most consistent in LAN, VIS1, VIS2, DAN, FPN, particularly during relational processing, working memory (2-back and 0-back), and story comprehension epochs. For instance, in the LAN during the story epoch, both amplitude and asymmetry were positively associated with task accuracy (Fig. 5d–f), with asymmetry explaining a slightly larger proportion of variance (R^2^ = 0.17; *d* = .28) than amplitude (R^2^ = 0.13; *d* = .24). These findings support the idea that increased engagement and lateralization of functional activity are beneficial for cognitive performance, particularly in tasks requiring language processing, attention, and memory. Higher-order polynomial models (up to the third degree) linking asymmetry to amplitude, and task accuracy to either amplitude or asymmetry did not improve model fit significantly over simpler linear models (DAIC < 3.7%; Fig. 5d–f), suggesting that the associations between these neural measures and behavioral performance are predominantly linear in nature.

### fMRI signal amplitude predicts task accuracy better than asymmetry

We also applied PLS regression, using within-sample 10-fold cross-validation, to predict task accuracy from either mean fMRI signal amplitude or interhemispheric asymmetry (Figs. 1e and 1g). Specifically, we used 153 predictors—mean amplitudes or asymmetry values within 9 major networks for 17 task epochs—to predict task accuracy across 12 task epochs: emotion (faces, shapes), gambling (reward, punish), language (story, math), relational processing (rel, match), social cognition (social, random), and working memory (0-back, 2-back). Prediction robustness was further validated using 2-fold cross-validation in the Discovery and Replication subsamples, as for asymmetry predictions from fMRI amplitudes.

**Figure 6:**
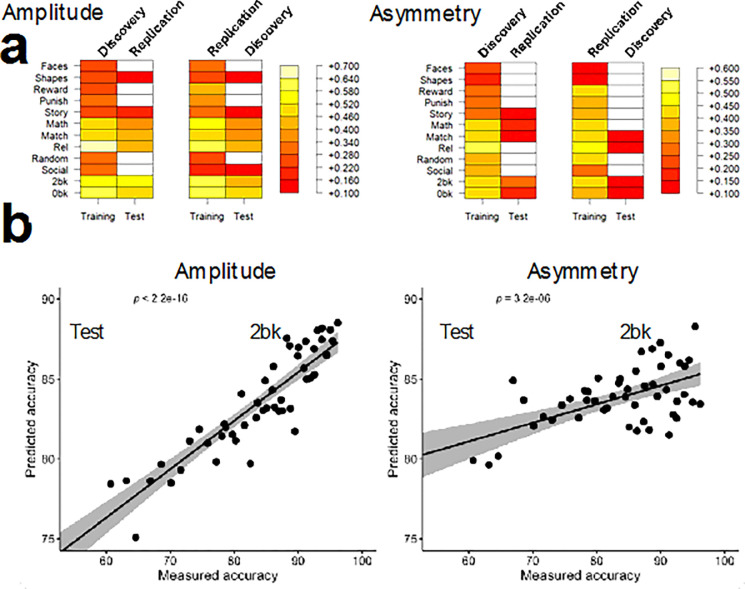
Prediction of task accuracy. (**a**) Significant correlations between measured accuracy and accuracy predicted from fMRI signal amplitude (left) and fMRI signal asymmetry (right) in the Training and Test samples, thresholded at P < 0.05 (Bonferroni-corrected for multiple comparisons). Predictions were derived using partial least squares (PLS) regression based on average fMRI measures within 9 major networks across 17 task epochs. (**b**) Representative scatter plots illustrating the linear association between measured and predicted accuracy in the Replication Test sample, shown for the 2-back epoch of the working memory task. Data are shown for Discovery (n = 504) and Replication (n = 485) subsamples.

As shown in Fig. 6a, both amplitude- and asymmetry-based models produced robust predictions of task accuracy, with consistent results observed in both Discovery and Replication samples. Prediction accuracy was highest for the 2-back, match, and the story epochs, particularly when using amplitude-based predictors. Although amplitude consistently outperformed asymmetry overall, asymmetry still accounted for significant variance in task accuracy across most conditions. The heatmaps also demonstrate strong generalizability, with prediction performance maintained from training to test data and across datasets. This is further illustrated in the scatter plots for the 2-back task (Fig. 6b), where amplitude-based predictions yielded a very strong linear association with measured accuracy (p < 2.2×10^−16^), and asymmetry-based predictions were also significant (p = 3.2×10^−6^), albeit with lower explained variance. These results suggest that both the strength and lateralization of task-evoked neural responses are meaningful contributors to inter-individual differences in cognitive performance, with amplitude offering a particularly strong and reliable signal for predictive modeling.

### Spatial components

**Figure 7:**
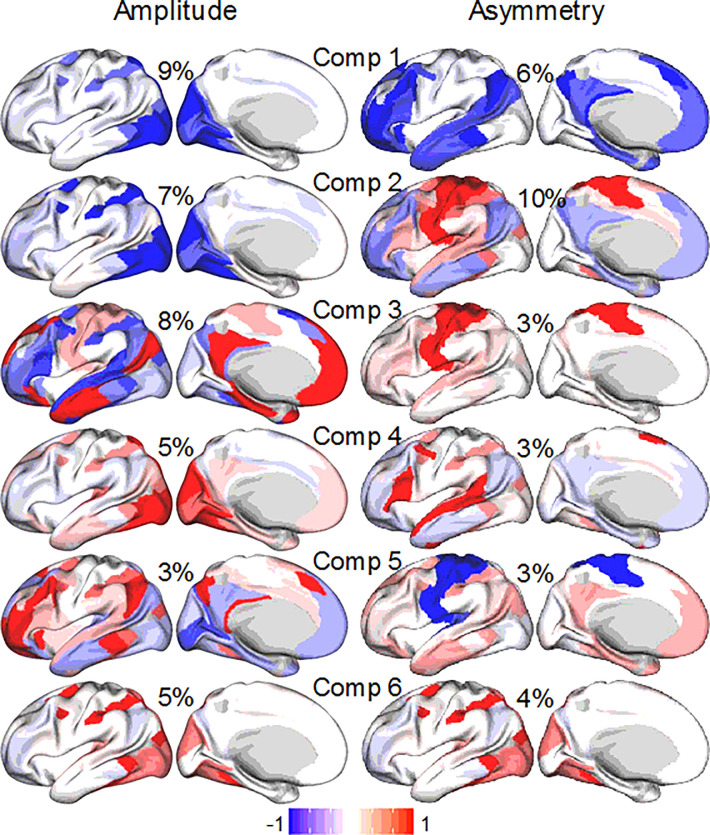
PLS components. The first six spatial components derived from partial least squares (PLS) regression models based on fMRI signal amplitude (left) or asymmetry (right), with corresponding explained variance (%) indicated. Results are from the discovery subsample (n = 504).

PLS regression revealed spatially distributed patterns across brain networks, with each component capturing distinct regions where either fMRI signal amplitude or asymmetry contributed to task-related variance in accuracy. The 12 primary components of fMRI signal amplitude captured 57% of the variance in the Discovery sample and 59% in the Replication sample. These components showed robust loadings in specialized networks, such as VIS1, VIS2, and DAN (components 1 and 2; Fig. 7). Additional components highlighted functional contributions from broader networks, including DMN and FPN, aligning with task demands across higher-order processing domains. Spatial maps for each component displayed consistent patterns across cross-validation folds, confirming that these PLS-derived networks are reproducible and stable predictors of task-relevant neural responses. For asymmetry, the 12 primary components captured 46% of the variance in the Discovery sample and 45% in the Replication sample, with prominent loadings in LAN and SMM. A combined PLS model incorporating both amplitude and asymmetry metrics did not outperform the amplitude-only model. This suggests that although asymmetry reflects distinct spatial features associated with task performance, its predictive contribution is secondary to that of amplitude.

### Effects of age and sex

PLS analysis of accuracy predictions based on fMRI signal amplitudes revealed significant effects of age and sex on component scores. Specifically, scores for component 2, which prominently loaded on VIS1, VIS2, and DAN networks, were higher in male compared to female participants and showed an age-related decrease across both Discovery and Replication samples (P < 0.003; Figure S6). For accuracy predictions based on asymmetry, PLS scores for components 1 and 3, which were heavily loaded on LAN and SMM networks, respectively, were significantly higher in female participants than in males (P<0.03; Figure S7).

## DISCUSSION

Here we examined the role of functional asymmetry in supporting task-specific processing across a range of cognitive domains, introducing a novel utilization of a laterality index applied directly to the cortical sheet. This approach enabled precise, vertex-wise measurement of lateralization across diverse functional networks, enhancing our understanding of the spatial patterns of brain asymmetry. Consistent with previous findings^9,12,13^, we observed distinct lateralization patterns associated with language, emotional processing, and motor control tasks, corroborating long-standing theories of hemispheric specialization^50^. Notably, all findings were highly reproducible across Discovery and Replication samples, underscoring the robustness of these lateralization patterns. Our results highlight the role of asymmetry in specialized processing, particularly in complex cognitive tasks, where distinct hemispheric contributions may help manage cognitive load and optimize performance accuracy. The observed robust lateralization patterns in networks supporting language, motor function, and social cognition align with prior large-scale analyses of functional lateralization that identified four principal axes of asymmetry—symbolic communication, perception/action, emotion, and decision-making—using meta-analytic mapping^17^.

Both greater fMRI signal amplitude and stronger hemispheric asymmetry were positively associated with task performance accuracy across multiple cognitive domains. These associations were especially robust in networks implicated in higher-order cognition and were most pronounced during relational processing, working memory, and story comprehension tasks. During the story comprehension task, asymmetry in the LAN network explained slightly more variance in performance than amplitude, suggesting a critical role for lateralized processing in language comprehension. The present findings suggest that both increased engagement (reflected in amplitude) and sustained hemispheric differentiation (reflected in asymmetry) contribute to better task outcomes, highlighting the functional relevance of maintaining both activation strength and lateralized organization across cognitive systems.

The left hemisphere model posits a dominant role of the left hemisphere in language processing, particularly in Broca’s and Wernicke’s areas, due to its involvement in verbal, logical, and sequential processing^51^. Our findings align with this model, showing marked leftward asymmetry in language, frontoparietal, and default-mode networks during Story epochs. This leftward specialization supports the predictive power of these networks in language tasks. On the other hand, the right hemisphere is more attuned to processing emotions, especially in recognizing social cues, facial expressions, and prosody^22^. Our findings from the emotion task, with observed rightward asymmetry in the DAN, FPN, and PMM networks, substantiate these theories. They suggest that right-hemisphere regions contribute to the automatic and holistic processing needed for emotional recognition and social cognition, a perspective in line with clinical observations of impaired emotional processing following right-hemisphere damage^52,53^.

The motor task results showing opposing asymmetries (rightward for left-side movements and leftward for right-side movements) in the somatomotor network underscore the well-known contralateral control of motor function^54^. This aligns with theories of sensorimotor lateralization, where contralateral processing supports efficient and precise control of movement such that each hemisphere is optimized for coordinating movements on the opposite side of the body^55^. It is also noteworthy that during the motor tasks we observed the expected lateralization in the SMM network with an equivalent lateralization in the DAN and AUD networks. This suggests an influence of motor behaviors in the asymmetry patterns of these two networks.

The observed asymmetry in relational processing and working memory tasks reflects the dual hemisphere contributions to these complex cognitive tasks, with the left hemisphere supporting more rule-based processing (i.e., relational processing task) and the right hemisphere providing spatial and contextual support (i.e., working memory task)^6^. Theories on lateralization in executive function propose that while the left hemisphere provides the computational basis for detail-oriented tasks, the right hemisphere handles broader contextual and visual-spatial processing, essential for working memory and flexible problem-solving^56^. The activation patterns in DMN and FPN networks during relational processing support this dual involvement, showing how each hemisphere may bring specialized contributions to comprehensive task integration.

Consistent with our hypotheses, the amplitude robustly predicted task accuracy across working memory, language, and relational processing tasks, suggesting that greater bilateral neural engagement in task-relevant networks translates to better performance. This relationship underscores that greater fMRI signal amplitude is not simply an indicator of neural activity but a meaningful predictor of task accuracy, suggesting that bilateral and higher engagement within task-relevant networks may enhance the brain’s functional capacity to support complex cognitive tasks. On the other hand, asymmetry was a more constrained predictor of task accuracy, with significant effects mainly in specifics epochs in working memory and relational processing tasks (only for 0- and 2-back, and match epochs). This observation suggests that hemispheric specialization provides targeted advantages in cognitive processes with limited capacity^57^, such as working memory, that necessitate efficient resource allocation to optimize performance. Hemispheric asymmetry, in this context, may help offset these constraints by dividing processing loads between hemispheres.

PLS scores for component 2 of the amplitude-based accuracy predictions, which were associated with VIS and DAN network engagement, were significantly higher in male participants compared to females and demonstrated a marked decline with age across both the Discovery and Replication samples. In contrast, for asymmetry-based accuracy predictions, scores for components 1 and 3, which were predominantly associated with LAN and SMM networks, respectively, were significantly higher in female participants compared to males. These findings suggest that age and sex may modulate the neural architecture underlying task performance, with a more bilateral engagement in males for amplitude-driven accuracy and a more asymmetric involvement in females across LAN and SMM networks. This sex-based divergence in neural organization could reflect distinct processing strategies that contribute to task performance across domains.

A potential limitation of our study is the restricted generalizability of the findings, given the narrow age range and inclusion of predominantly healthy, young adult predominantly White sample drawn from the Human Connectome Project (HCP); this should be explicitly acknowledged, as lateralization patterns and their associations with cognitive performance may vary substantially across different age groups or clinical populations. Furthermore, our study uses a cross-sectional approach, which limits the ability to infer causal relationships between amplitude, asymmetry, and cognitive performance.

Our study supports the hypothesis that functional asymmetry contributes to performance accuracy by enabling efficient, specialized processing across cognitive domains. However, we also found that mean fMRI signal amplitude was a stronger and more consistent predictor of task performance, suggesting that while lateralization provides a structural and organizational scaffold for specialization, it is the degree of neural engagement—reflected in amplitude—that more directly supports performance. These findings are not contradictory but rather point to a complementary relationship: asymmetry may shape the architecture for task-relevant processing, whereas amplitude reflects dynamic, state-dependent modulation of neural resources. Future research might examine how this interplay between asymmetry patterns and flexible amplitude responses informs individual differences in cognition and clinical interventions targeting disrupted lateralization, such as in depression and schizophrenia.

## METHODS

### Subjects

The data used in this study were sourced from the publicly accessible WU-Minn HCP 1200 Subjects data release (http://www.humanconnectome.org/). The scanning protocol was approved by the Human Research Protection Office (HRPO) at Washington University in St. Louis. All participants included provided written informed consent. One-hundred and sixty-one participants were excluded from the study due to incomplete image datasets. The remaining 989 participants (age: 28.8 ± 3.7 years; 607 females) were included in this study. No experimental procedures involving human subjects were conducted at the author’s institutions. Participants were divided into Discovery (n = 504) and Replication (n = 485) subsamples matched by sex, age, and BMI using the sample.split function of the caTools R library.

### Task-fMRI paradigms

The HCP fMRI tasks paradigms are thoroughly detailed elsewhere^58^. They encompass a diverse set of cognitive domains, including motor, language, social cognition, relational processing, working memory, gambling, and emotion processing, designed to probe distinct neural networks and capture the functional specialization and integration of the human brain (https://www.humanconnectome.org/hcp-protocols-ya-3t-imaging):

1. In the *emotion* task^59^, participants matched either the emotional expression (angry or fearful) of a face at the top of the screen to one of two faces at the bottom or matched shapes in a similar manner. Each run contained 3 face epochs and 3 shape epochs, with 6 trials per epoch. Epochs were preceded by a 3 -second cue and lasted 21 seconds, with stimuli presented for 2 seconds and a 1-second ITI. Each run consisted of six blocks and had total duration of approximately 2 minutes and 16 seconds.
2. In the *gambling* task^60^, participants played a card-guessing game where they predicted if the number on a mystery card (1–9) was higher or lower than 5 to win or lose money. They made their guess by pressing a button, and feedback (a green up arrow with “$1” for wins, a red down arrow with “-$0.50” for losses, or a neutral arrow for neither) was provided for 1 second. Each run contained 2 “mostly reward” and 2 “mostly loss” epochs (28 seconds each), with 8 trials per epoch, and 4 interleaved fixation epochs (15 seconds each). Each run of the task has a duration of approximately 3 minutes and 12 seconds.
3. The *language* task^61^ includes 4 epochs of a math task and 4 epochs of a story task in each of two runs. Story epochs presented short auditory stories (5–9 sentences), followed by a forced-choice question about the story. The math task, designed to match the length of the story epochs (~ 30 seconds), involved solving auditory arithmetic problems. The math task was adaptive to ensure a consistent difficulty level across participants. Each run of the task has a duration of approximately 3 minutes and 57 seconds.
4. The *motortask*^54,62^ involves visually cued movements like tapping fingers, squeezing toes, or moving the tongue. Each epoch lasted 12 seconds and was preceded by a 3-second cue. Each run included 2 epochs for each movement type (tongue, right hand, left hand, right foot, left foot) and 3 fixation epochs (15 seconds each). Each run of the task has a duration of approximately 3 minutes and 34 seconds.
5. In the *relational processing* task^63^, participants viewed two pairs of objects, each with a distinct shape and texture. One pair was displayed at the top and the other at the bottom of the screen. During “relation” epochs, participants identified which feature (shape or texture) differed in the top pair, then determined if the bottom pair differed along the same dimension. During control “match” epochs, participants compared two objects at the top with one object at the bottom, using a word cue (either “shape” or “texture”) to decide if the bottom object matched either of the top objects in that dimension. Stimuli were shown for 2800 ms, with a 400 ms inter-trial interval (ITI), and each epoch consisted of 5 trials. Each of the two task runs contained 3 “relation,” 3 “match,” and 3 “fixation” epochs, each lasting 16 seconds. Each run of the task has a duration of approximately 2 minutes and 56 seconds.
6. In the *social cognition* task participants watched 23-second video clips of shapes (squares, circles, triangles) either interacting socially or moving randomly^64,65^. After each clip, participants judged whether the movements reflected social interaction, uncertainty, or no interaction. Each run included 5 video epochs (either social or random) and 5 fixation epochs (15 seconds each). Each run of the task has a duration of approximately 3 minutes and 27 seconds.
7. The *working memory* task (1) presents 4 categories of images (faces, places, tools, and body parts) across 8 epochs in 2 runs. Each category had a 0-back epoch (press a button for a target stimulus) and a 2-back epoch (press a button when the current stimulus matches one presented two steps earlier). Each epoch lasted 27.5 seconds and included ten 2.5-second trials, with 2 targets and 2–3 non-target lures (stimuli repeated in incorrect n-back positions). Each stimulus was displayed for 2 seconds, followed by a 500 ms ITI. Each run of the task has a duration of approximately 5 minutes.

### fMRI data

The fMRI data acquisition and image preprocessing methods of the HCP are described elsewhere^47,66^. Briefly, functional imaging was conducted using a 3.0T Siemens Skyra scanner (Siemens Healthcare, Erlangen, Germany) equipped with a 32-channel coil. The imaging protocol employed a gradient echo-planar imaging (EPI) pulse sequence with a multiband factor of 8, a repetition time (TR) of 720 ms, an echo time (TE) of 33.1 ms, a flip angle of 52°, a matrix size of 104 × 90, and 72 slices, producing isotropic voxels of 2 mm^67,68^. Scans were collected using both left-right (LR) and right-left (RL) phase encoding directions to enhance data reliability. Preprocessing included gradient distortion correction, rigid-body realignment, field-map processing, and spatial normalization to the Montreal Neurological Institute (MNI) stereotactic space^47^. For the analysis, we utilized individual cross-run first-level contrasts of parameter estimates in CIFTI format from the WU-Minn HCP 1200 Subjects Data Release. We used 2 contrasts from the emotion task (faces and shapes, totaling 1,978 images), 2 from the gambling task (reward and punish, 1,978 images), 2 from the language task (story and math, 1,978 images), 5 from the motor task (left hand, right hand, left foot, right foot, and tongue, totaling 4,985 images), 2 from the relational processing task (rel and match, 1,978 images), 2 from the social cognition task (social and random, 1,978 images), and 2 from the working memory task (0-back and 2-back, 1,978 images). Notably, the use of fMRI signals in the symmetric CIFTI grayordinate space enabled vertex-wise correspondence between hemispheres and facilitated analyses of functional asymmetry.

### fMRI asymmetry index

A normalized index of asymmetry was calculated by subtracting BOLD signal estimates in the right (R) hemisphere from those in the left (L) hemisphere for each task, contrast, and individual, following the formula:

[1] $$\Delta =\frac{L\;-\;R}{\left|L|\;+\;|R\right|}\;.$$

The denominator, |L|+|R|, represents the bilateral BOLD signal amplitude and reflects the combined activation from both hemispheres. This approach allowed for the quantification of asymmetry while accounting for the magnitudes of the estimates in both hemispheres^43^. A positive value of the asymmetry index indicates greater activity in the left hemisphere compared to the right, suggesting a leftward lateralization of the measured function. Conversely, a negative value suggests greater activity in the right hemisphere, indicating rightward lateralization. The correspondence of 32,492 vertices in the left hemisphere with those in the right hemisphere was established through correlation analysis of the Cartesian coordinates for each vertex, relative to the center of the bounding box (*R*>0.995)^43^. This threshold-independent index on the cortical sheet provides a more nuanced view of interhemispheric lateralization, capturing subtle asymmetries that may be lost with threshold-dependent indices in the volumetric Cartesian space.

### Partial Least Squares (PLS) Analysis

PLS regression projects both the predictor **X** and response **Y** matrices into lower-dimensional spaces and creates latent variables which represent linear combinations of the original variables while retaining the most meaningful variability in the data. By focusing on latent variables, PLS bypasses issues with highly correlated predictors, and can handle situations where the number of predictors is larger than the number of observations. Furthermore, the latent variables can be interpreted as meaningful patterns in the data.

PLS regression was conducted in R to examine the relationships between fMRI signal amplitude, asymmetry, and task accuracy. First, individual contrasts corresponding to 17 task epochs (faces, shapes, reward, punish, story, math, left hand, right hand, left foot, right foot, tongue, rel, match, social, random, 0-back, and 2-back) were averaged within 9 major networks (VIS1, VIS2, DMN, DAN, FPN, LAN, CON, AUD, SMM) to construct two matrices with 153 regressors, one for fMRI signal amplitude (**B**) and the other for asymmetry (**A**). Then, individual accuracy scores corresponding to faces, shapes, reward, punish, story, math, rel, match, social, random, 0-back, and 2-back epochs were arranged in another matrix with 12 variables (**C**). For amplitude-based predictions of asymmetry we set **X** = **B** and **Y** = **A**. For predictions of accuracy based on amplitude or asymmetry, we set **Y** = **C** and **X** = **B** or **A**.

The ORA, VMM, and PMM networks were excluded from the PLS prediction model due to their relatively small size and the diffuse, less task-specific nature of their activation patterns. As a result, including these networks could introduce significant variability and reduce the model’s predictive accuracy. By prioritizing reliable networks with more distinct and localized functional roles, we aimed to increase the model’s sensitivity to task-specific effects and improve the interpretability of the results.

### Statistical analyses

Vertex-wise statistical analyses of fMRI signal asymmetry were conducted in MATLAB 2022a. To account for potential confounds, we first regressed out age, using linear regression, followed by grand mean scaling to normalize the data across categorical variables (sex and race). The statistical significance of asymmetry was then assessed using vertex-wise t-tests, with a threshold p < 0.05, false discovery rate (FDR) corrected for multiple comparisons across 32,492 vertices on the cortical sheet. Statistical analyses within specific regions of interest (ROIs) we conducted using t-test in R. To compare the fit of linear and nonlinear models, we computed the Akaike Information Criterion (AIC) for each model, with lower AIC values indicating a better fit to the data. This approach allowed us to assess whether the addition of nonlinear terms, such as higher-order polynomials, provided a significant improvement over the simpler linear models.

## Data Availability

HCP data are publicly available through the ConnectomeDB data management platform (https://db.humanconnectome.org/).
